# The Role of Talker Information in Phonetic Convergence

**DOI:** 10.1177/17470218261442873

**Published:** 2026-04-03

**Authors:** Orhun Uluşahin, Elhaam Parveen Hasan, Hans Rutger Bosker, Antje S. Meyer, James M. McQueen

**Affiliations:** 1 Max Planck Institute for Psycholinguistics, Nijmegen, The Netherlands; 2 International Max Planck Research School for Language Sciences, Nijmegen, The Netherlands; 3 Faculty of Science, Radboud University, Nijmegen, The Netherlands; 4 Donders Centre for Cognition, Radboud University, Nijmegen, The Netherlands

**Keywords:** speech perception, talker information, speech production, phonetic convergence, representational parity

## Abstract

The nature of the link between speech perception and production is extensively debated. One question in this debate concerns the possible use of shared representations. Talker representations encode information about particular talkers’ speech and are formed through perception. We present three experiments that investigate the potential role of talker representations in speech production as measured by their impact on phonetic convergence to voice fundamental frequency (F0). In Experiment 1, female native Dutch speakers (*N* = 32) performed a baseline reading task, followed by a synchronous speech task where they were instructed to synchronize (temporally) to a pre-recorded model talker. The model talker’s voice was pitch-shifted to have high or low F0, and half of the sample performed the task in each F0 condition. Participants’ F0 values converged toward that of the model talker. In Experiment 2, we added an exposure task before synchronous speech, during which participants (*N* = 32) were familiarized with the model talker at high or low F0. At test, they heard the model talker at the familiar F0. Finally, in Experiment 3, we reversed the F0 manipulation between exposure and test. Thus, participants (*N* = 32) were familiarized with either high or low F0, but heard the opposite F0 condition during synchronous speech. The data revealed that while congruent and conflicting talker information both diminish convergence magnitude, only conflicting talker information reduces the probability of convergence. These results indicate that talker information acquired exclusively through perception is used during production, suggesting shared representations across perception and production.

## Introduction

Speech perception is significantly improved when the listener already knows something about how the talker speaks. Familiar talkers’ voices are easier to distinguish in noise (Nygaard et al., 1994; Nygaard & Pisoni, 1998) and generally more intelligible (Holmes et al., 2021), even when the listener may not be able to explicitly recognize the voice as being familiar (Holmes et al., 2018). However, despite knowledge of these facilitatory effects of prior talker information in speech perception, research into the potential role of talker-specific speech information in speech production is limited, despite the potential of such research to reveal valuable insights into the intricate link between language perception and production. To that end, this study tests whether the presence of talker information leads to observable changes in production observed through the impact of conflicting versus congruent talker information on the probability and convergence magnitude.

This potential role of talker information in production speaks to an ongoing theoretical debate concerning the cognitive architecture of language processing. Relatively recent integrative theories (e.g., McQueen & Meyer, 2019; Pickering & Garrod, 2013) argue for a unified language system that dynamically (re-)uses resources, instead of separate production and perception systems that mostly or exclusively use resources that may not be accessible to the other system. Many influential models of perception (e.g., TRACE; McClelland & Elman, 1986; Shortlist B; Norris & McQueen, 2008) and production (e.g., Levelt, 1999; Levelt et al., 1999) focus on task-specific resources, demands, and/or representations. Talker representations (i.e., mental representations encoding information about individual talkers’ speech) present an interesting opportunity to test these two types of theory. Talker representations are formed exclusively through perceptual processes, by exposure to a new talker, and are used ubiquitously in speech perception. There is evidence that listeners encode different talkers’ speech features, including acoustic features of speech sounds (e.g., Reinisch, 2016; Nygaard & Pisoni, 1998; Zhang & Holt, 2018), as well as talkers’ lexical choices (e.g., Trainin & Shetreet, 2024). These features are extracted from incoming speech from a given talker, abstracted, and stored for long durations. Despite this extensive knowledge about the role of talker representations in perception, the lack of research into the potential role of talker information in language production remains a major gap in the current literature.

In addition to evidence for the tracking and long-term storage of talker information at the sound level, accumulating evidence also clearly indicates that acoustic features such as fundamental frequency (e.g., Babel & Bulatov, 2012; Bradshaw & McGettigan, 2021; Rahimi et al., 2017), speech rate (e.g., Bell et al., 2003; Casasanto et al., 2010; Schultz et al., 2016), segmental features such as voice onset time (Nielsen, 2011) and vowel formants (Bradshaw et al., 2025; Pardo et al., 2013), as well as higher-level aspects of speech such as syntactic structures (e.g., Branigan et al., 2000, 2007) and word choices (e.g., Branigan et al., 2011; Brennan & Clark, 1996; Cai et al., 2021; Garrod & Anderson, 1987) are modeled by listeners, who also gradually liken their speech to that of their interlocutor over the course of the linguistic interaction. This phenomenon is referred to by multiple names (e.g., *convergence* [which this manuscript uses], *alignment*, *entrainment*, *(speech) accommodation*, *(phonetic/speech) imitation*, *(speech) priming*), and it represents a clear interaction of perceptual and productive processes. If these processes do not (at least partially) rely on shared representations, this necessitates duplicate or analogous representations across the cognitive architecture of language perception and production. Integrative theories (e.g., Pickering & Garrod, 2013) view convergence as a direct (and inevitable) consequence of the automatic integration of perception and production tasks. According to this view, they rely on shared representations to cut processing costs, allowing one’s interlocutor(s) to take on part of the cognitive load associated with conversational tasks. For instance, one might simply repeat a word that an interlocutor has used, as opposed to retrieving a synonym that may be more familiar to oneself or more frequent in general. While integrative theories acknowledge a social function for convergence without asserting its centrality, an alternative view (e.g., C. S. Kim & Chamorro, 2021; Pardo et al., 2012, 2018; Schweitzer & Lewandowski, 2014) interprets convergence as a primarily social phenomenon. These studies often cite failures to demonstrate the automaticity prescribed in integrative theories, as social factors outweigh various linguistic manipulations in predicting linguistic output. One convenient way to test this automaticity involves investigating whether representations might be shared across perception and production. Since talker representations are formed through perceptual processes alone, the potential role of talker information thus provides an opportunity to explicitly test the automatic integration of perception and production.

So far, the role of talker information (and its reliability) in convergence has only been investigated indirectly. For instance, one study has looked into convergence in college roommates (Pardo et al., 2012), emphasizing the social function of convergence and the social factors that influence it, rather than deliberately manipulating talker *information as defined by prior knowledge about the* acoustic-phonetic profile of a given talker. In the present study, we investigate whether perceptual and productive language tasks (at least partially) rely on the same talker representations, using acoustic measures to investigate potential changes in participants’ speech as a function of exposure to a manipulated model talker. Crucially, our definition of talker familiarity better fits into a framework of listener information about a talker’s speech characteristics and the reliability of these acoustic-phonetic cues.

The precise effects of talker *familiarity* on the perception and production of particular acoustic features remain debated. However, reviews of convergence studies using multiple acoustic metrics (e.g., Pardo et al., 2017) highlight that convergence to duration and F0, especially over larger units of speech (e.g., sentences instead of isolated phonemes) is documented more consistently than convergence to formants or other acoustic features. In line with this observation, recent speech production studies have also demonstrated that convergence to sentence-level mean F0 can be reliably observed in experimental settings. In one online study (Bradshaw & McGettigan, 2021), participants first read 50 sentences to provide baseline F0 values. Later, the same participants read 50 sentences with a *model talker* (referred to as an “accompanist” by the authors) in a synchronous speech task. In the synchronous speech task, participants were instructed to try to synchronize (i.e., temporally) with a recording by the model talker, to the best of their ability. While participants were led to believe that the experiment was about synchrony in the temporal domain, this design allowed them to manipulate other acoustic variables. In this case, the model talker’s F0 was manipulated, and participants’ F0 values were measured. The sample was assigned to high F0, low F0, and extra-low F0 groups, with 10 randomly assigned participants in each group. The model talker’s voice in the synchronous speech task had been manipulated by +2, −5, and −9 st (semitones) for the high, low, and extra-low F0 conditions, respectively. Participants’ mean F0 measures across the synchronous speech task were compared to their baseline measures from the reading task. The results revealed that participants typically followed one of three patterns: *Convergence*, whereby the participant’s F0 moved in the direction of the accompanist’s; *divergence*, whereby the participant’s F0 moved away from the accompanist’s; and *non-convergence*, whereby the participant’s F0 was unaffected by the F0 manipulation present in the accompanist’s voice (i.e., no significant difference between reading and synchronous speech). Crucially, the majority of the sample (~60%) exhibited convergence whereby their F0s became more like the model talker’s during the synchronous speech task. In a second experiment, speech was elicited using an audio-only condition (i.e., same as in the first experiment), an audiovisual condition (i.e., participants read the sentence while watching a video of the accompanist reading the same sentence), and a visual-only condition (i.e., participants read the sentence while watching a silent video of the accompanist reading the sentence). Convergence was only observed in the two conditions that included audio signals during trials, suggesting that participants’ productive F0 changes were indeed modeled on the accompanist’s speech instead of their articulatory gestures or visual cues.

In another study (Aubanel & Nguyen, 2020), pairs of participants in two sound-attenuating booths took turns reading sentences from the same text. A computer manipulated participants’ mean F0 values in real time to match the F0 profile over trials to a sine wave pattern pre-defined by the researchers. That is, over a period of 74 trials, each trial’s F0 was manipulated “live” and streamed to the listening participant, who became the reader for the following trial. Thus, the precise F0 values over the 74 trials were a sinusoidal shape. Although the F0 differences across any two consecutive trials were below the just noticeable threshold for F0, participants were generally able to follow the F0 changes, mimicking the sinusoidal F0 profile across the task, and converging to each other’s perceived speech, despite the reading-based nature of the task. Thus, both consistent exposure to static (but manipulated) F0 values and exposure to rapidly changing F0 values across minimally different consecutive trials have been demonstrated as facilitating convergence to mean sentence F0, indicating that listeners can converge to F0 implicitly in controlled laboratory settings.

These studies suggest that F0 is a reliable dependent variable, but also that the mode of instruction in laboratory convergence experiments is a critical design choice. The results of studies investigating phonetic convergence in conversational settings often do not align with the reports from those investigating convergence in tightly controlled lab settings, although both types of studies can detect convergence (Pardo et al., 2017, 2018). If experiment instructions include explicit demands for a participant to “repeat” or “imitate” acoustic stimuli, the automaticity that forms the core of theories of integrative theories becomes difficult to establish. The synchronous speech task used by Bradshaw and McGettigan (2021) affords both extensive experimental control and a reliable way to measure convergence on acoustic grounds without explicit instructions to converge to the target acoustic feature. The participants are told that the experiment is about timing/synchrony (i.e., a perfectly plausible subject for a psycholinguistic experiment) while the experimenter gains the freedom to manipulate and measure many acoustic variables other than temporal features.

To investigate the potential role of talker information on speech production, and its implications on the link between perception and production, the present study presents three experiments which targeted convergence to mean sentence F0 in three different ways: (a) Without prior talker F0 exposure, (b) with congruent talker F0 exposure prior to synchronous speech (i.e., familiarization with a high/low F0 model talker who is heard at the same mean F0 throughout the experiment), and (c) with conflicting talker F0 exposure prior to synchronous speech (i.e., familiarization with a high/low F0 model talker who is heard at the opposite F0 condition across exposure and test). In all three experiments, we obtained reading baseline values from participants in a reading task. In Experiment 1, we manipulated the mean F0 of a model talker’s Dutch sentences in a synchronous speech task to assess whether and how participants converge to the F0 profile of the model talker. In Experiment 2, we investigated the effect of talker information by adding an exposure phase to the paradigm introduced in Experiment 1. Here, participants were first familiarized with a high or low F0 model talker, then they performed a synchronous speech task where they heard consistent F0 information from the same talker. Finally, in Experiment 3, we once again familiarized two groups of participants with high or low F0, respectively, but changed the test (i.e., synchronous speech task) to include conflicting talker information. That is, participants who were familiarized with low F0 heard high F0 during synchronous speech, and vice versa. It is worth noting that while this reversal may come at the cost of ecological validity (i.e., talkers typically do not change their average F0 by 4 st), it affords excellent experimental control with respect to our research question as it allows us to maintain all other aspects of the model talker’s speech except the F0.

Given the evidence discussed above, we expect the majority of participants in all three experiments to display measurable convergence (i.e., a significant departure from their reading baseline as a function of exposure to our speaker, in the direction of the model talker’s target F0). Crucially, in line with a hypothesis of shared representations across perception and production, we expect talker familiarity to have an observable impact on the probability and/or the magnitude of convergence. A number of potential outcomes follow from this overarching hypothesis: Talker representations, though formed exclusively through exposure to the model talker, can facilitate or hinder convergence. Facilitation is more likely in the case of congruent talker information (Experiment 2), and hindrance is more likely in the case of conflicting talker information (Experiment 3). While the former outcome could be attributed to an improved ability to extract acoustic detail from the speech signal of a familiar talker, the latter outcome could be attributed to increased conflict in the perceptual stream (i.e., due to the conflicting input) manifesting in production. We avoid taking a strong stance on whether qualitatively different facilitatory or inhibitory mechanisms (or a combination) exist within this framework, and aim to inform these issues through the experiment series presented here.

## Experiment 1 – No Prior Talker Information

We first ran an experiment without a talker information manipulation to establish baseline convergence metrics within a synchronous speech paradigm. Thus, Experiment 1 functioned as a stepping stone for Experiments 2 and 3, and was a conceptual replication of the audio-only portion of Bradshaw and McGettigan (2021).

### Method

#### Participants

Thirty-two native Dutch speakers (age range: 20–32, *M*_age_ = 24.25, *SD*_age_ = 3.08) participated in the experiment. To ensure that participants’ articulatory F0 ranges generally aligned with the (female) model speaker’s, we recruited only female participants. All participants reported having normal or corrected-to-normal vision, no speech impairments, and no auditory impairments. All participants received monetary compensation for their participation. Participants were randomly distributed into two even groups: high F0 and low F0. This assignment simply determined the F0 of the stimuli they would encounter in the synchronous speech task, and was not based on the participants’ own F0. All experiments presented in this manuscript were approved by the Ethics Committee of the Faculty of Social Sciences of Radboud University (project code: ECSW-2019-019).

#### Materials

For all auditory stimuli, we used recordings from the same female native speaker of Dutch, recorded in one session using one set of configurations. We recorded a total of 85 sentences for the experiment (see “sentences.txt” on OSF for a complete list of sentences). These 85 sentences were divided into three sets: Sets (A) and (B) consisted of 40 sentences and were used in the reading and synchronous speech tasks. Set (C) consisted of five sentences and was used in the practice trials for the synchronous speech task. All sentences were minimally 10 words (average = 12 words, maximum = 15 words) and 63 characters (average = 72 characters, maximum = 88 characters) long to ensure that they could not be fully read during the 3-s countdown at the start of each trial (see section “Procedure” below).

After recording, sound files were manually cropped such that the onset of the first phoneme of each sentence began no later than 5 ms into the files, ensuring that the start of playback would closely align with the start of spoken stimuli. These cropped audio files were then pitch-shifted using PSOLA in Praat (Boersma & Weenink, 2022). Based on F0 baselines obtained from a pre-test of 10 female native speakers of Dutch, we set the reference mean F0 level at 210 Hz. Then, to create the stimuli for the high F0 and low F0 conditions, we pitch-shifted each audio file by +2 and −2 st, respectively, relative to 210 Hz (i.e., files were not first shifted to 210 Hz, but the shifting target was determined based on this value). Given the 85 sentences recorded for the experiment, 170 manipulated sound files were used in the experiment, with a set of 85 sentences at ~236 Hz mean F0 (+2 st), and another 85 at ~187 Hz (−2 st).

We opted to use 40 sentences for the baseline reading task and 80 for synchronous speech primarily for reasons of statistical power. We operated on the empirical precedent established by Bradshaw and McGettigan (2021) and assumed 40 sentences would be enough data to obtain reliable baseline F0 values. We then doubled this number in synchronous speech to approach convergence metrics which we assumed would display greater variation given expected differences in participants’ responsiveness to the F0 manipulations.

#### Procedure

After providing informed consent, participants proceeded to a sound-attenuating booth equipped with stereo headphones, a keyboard, a 24-inch screen, and a directional condenser microphone placed ~60 cm away from the participant.

In the reading task, half of the participants within each F0 group saw sentences only from Set A, and the other half saw sentences from Set B. In each trial, a sentence appeared on screen along with a 3-s countdown. At the end of the countdown, the countdown was replaced by the caption *OPNAME* “RECORDING.” Participants were instructed to start speaking only after the caption appeared, and had 9 s to read the full sentence (i.e., each trial lasted a maximum of 12 s). Despite this, recording for each trial actually began at the trial onset (i.e., when the countdown began). Participants also had the option to terminate the trial by a button press after speaking. To mitigate potential early interruptions at sentence-final word onsets, recording continued for 500 ms after the button press. After a second-long inter-stimulus interval, the next trial began. After completing all 40 trials, participants moved on to the practice trials for the synchronous speech task.

The practice trials for the synchronous speech task were explicitly introduced to the participants as an opportunity to learn about the speaker’s speech rate, and participants were informed that data from this task would not be analyzed. The trial structure differed from the reading task in a single but significant way: At the end of the 3-s countdown, the model talker’s recording of the sentence assigned to the trial was played. The mean F0 of the model talker’s utterance was determined by the participant’s assigned F0 condition. The sentences used in the practice trials (Set C) were not used elsewhere in the experiment. Participants were instructed to try their best at synchronizing with the model talker (i.e., temporally) with no explicit instruction to imitate or otherwise model any other acoustic feature.

Upon completing the five practice trials, participants proceeded to the synchronous speech task. The trial structure of the synchronous speech task was identical to that of the practice trials. Each participant encountered all 80 non-practice sentences (i.e., Set A + Set B) in one of eight pseudorandomized trial lists. In all of these lists, no more than two consecutive trials belonged to the same set. Thus, participants did not encounter “blocks” of sentences they had previously seen in the reading task. Instead, novel sentences and previously encountered sentences were evenly distributed across the experiment. As with the reading task, the recording in the synchronous speech task began at trial onset and ended 500 ms after participants’ key presses, with a timeout set to 12 s. A second-long inter-stimulus interval separated the 80 trials.

After the synchronous speech task, participants completed a post-experiment questionnaire (see “questionnaires” on OSF), which marked the end of the experiment.

#### Extracting Acoustic Information From Participant Responses

As the first step of our analyses, we used a custom Praat script (see section “OSF”) to extract F0 and intensity values from our participants’ recorded responses. For the reading task, we used a relatively wide range for pitch tracking in typical female speakers (i.e., 120–400 Hz) and we extracted F0 information from all voiced segments of a recording. Given that synchronous speech trials began with a 3-s countdown, and that the longest pre-recorded sentence lasted no more than 3.8 s, we extracted F0 information only from the 2.8 to 7 s period of each recording. For pitch tracking, we set the lower and upper boundaries an octave below and above the per-participant mean F0 observed in the reading task. Thus, any variation within an octave of each participant’s baseline could be reliably detected.

### Results

**Figure 1.**
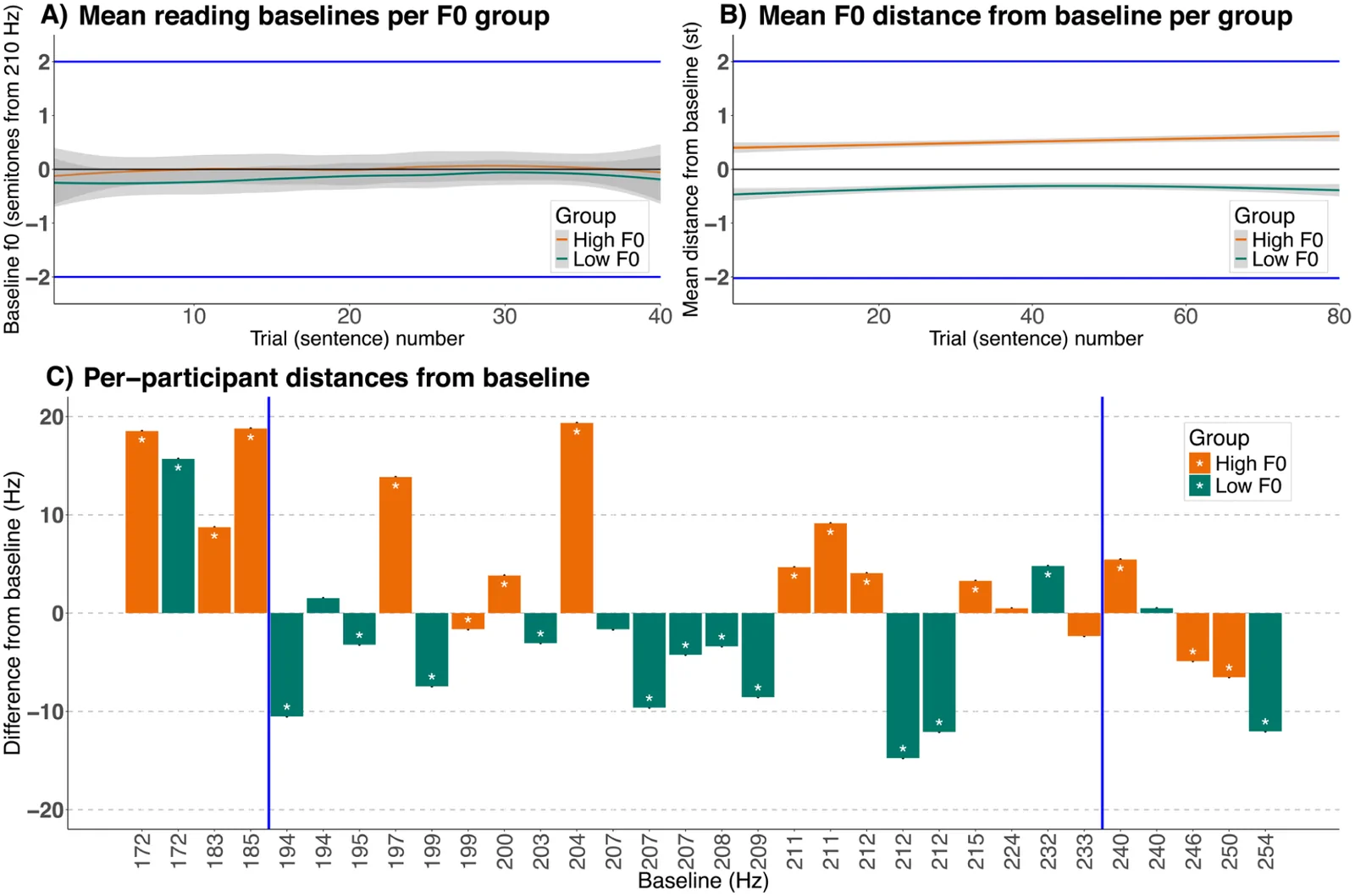
Reading F0, synchronous speech F0, and convergence metrics for Experiment 1. (A) Mean reading baselines per F0 group. (B) Mean F0 distance from baseline per group. (C) Per-participant distances from baseline. *Note.* Panel A demonstrates the comparable baselines across the two F0 groups in Experiment 1. On average, participants from both the high F0 and low F0 groups had baselines within a few Hz of the pre-tested mean of 210 Hz. Panel B demonstrates the changes in F0 in the synchronous speech task in Experiment 1. Here, the values are relative to each participant’s baseline, allowing for an overview of participant-normalized measures of changes in the direction of the model talker. Panel C displays convergence metrics on a by-participant basis. The *x* axis is ordered by participant baseline, but not scaled. Thus, each bar corresponds to a participant, with F0 group indicated by bar color (i.e., orange => high F0, green => low F0). The two vertical blue lines indicate the model talkers’ mean F0 values for the two F0 conditions (i.e., ~187 and ~236 Hz for the low and high F0 conditions, respectively). Bars with asterisks mark participants whose F0 changes from reading to synchronous speech were significant. In a perfect convergence scenario, all participants below the low F0 target would increase their F0, all above the high F0 target would lower it, and those whose baselines fall between the two target F0 values would move in the direction of their condition’s F0 target. In Experiment 1, based on the criteria described below, 24 out of the 32 participants converged.

All analyses, unless otherwise noted, were conducted using R (R Core Team, 2022). In all experiments reported in this manuscript, data from trials in which participants took <2,500 ms to terminate a synchronous speech trial were excluded, since we took this as reflecting a failure to comply with task instructions for a given trial. If a button was not pressed but the trial timed out with a valid F0 value, trial data were kept. In Experiment 1, only two trials were excluded based on these criteria. A summary of the results is visualized in Figure 1.

#### Analyses of Convergence

##### Modeling Convergence in Raw F0 Across Tasks

We first modeled F0 across the two tasks, across the two F0 conditions, and across all participants with a single linear mixed-effects model (The Low Model; with the low F0 group mapped the intercept), using the R package “lmerTest” (Kuznetsova et al., 2017). With F0 as the dependent variable, we included fixed effects of Task (i.e., reading or synchronous speech), Condition (i.e., high F0 or low F0), and an interaction between them. Both were coded as categorical predictors, with the low F0 condition and the reading task mapped onto the intercept. The model also included random intercepts for Item (i.e., sentence) and Participant. Random slopes were omitted to address convergence issues.

Within The Low Model, Task significantly predicted F0 (β = −4.26, *SE* = 0.33, *z* = −12.84, *p* < .001) whereas Condition did not (β = 2.25, *SE* = 6.38, *z* = 0.35, *p* = .73). Given that the low F0 condition and the reading task were on the intercept, the absence of a Condition effect confirmed our assumption that F0 baselines were comparable across the two groups. The significant effect of Task indicated that participants in the low F0 condition had significantly lower F0s in the synchronous speech task compared to their baseline F0s during reading. In addition, we observed a significant interaction between Task and Condition (β = 10.18, *SE* = 0.47, *z* = 21.71, *p* < .001). This prompted the releveling of the model such that the high F0 condition and the reading task were now mapped onto the intercept. Within this model (The High Model; with the high F0 group mapped the intercept), Task significantly predicted higher F0 (β = 5.92, *SE* = 0.33, *z* = = 17.87, *p* < .001) while the results for Condition and the interaction between Task and Condition were identical to those of The Low Model in all aspects except sign. Thus, over the course of the experiment, participants in both groups reliably converged away from their baselines, in the direction of the pitch manipulation they encountered in the synchronous speech task. Output tables for these models are available in Appendix A (in the >Supplemental Material) and on OSF (under “analyses/exp_1”).

##### Within-Subject Analyses of Convergence

We used participant-by-participant Analyse﻿s of Variance (ANOVAs) to divide the sample into convergers and non-convergers. Convergence was assessed on the basis of two simultaneous criteria: (a) If a participant’s synchronous speech task mean F0 was significantly different from their mean F0 in the reading task and (b) if this change was in the direction of the target talker, the participant was designated as a converger. The latter criterion thus allowed us to correctly detect convergence also in cases where a given participant’s baseline F0 might be above the high F0 target or below the low F0 target. Based on these two criteria, 24 participants converged, 2 diverged, and 6 displayed no significant differences in their F0 production across tasks.

#### Secondary Analyses

We ran several secondary analyses to confirm assumptions regarding our experimental design and to check for possible confounds. Further details regarding these secondary analyses are provided on OSF for all three experiments.

##### Baseline Homogeneity and Representativeness Across Groups

In addition to the results of The Low Model, a one-way ANOVA across all reading task trials revealed no significant differences in baseline F0 measures between the two F0 groups [*F*(1, 30) = 0.091, *p* = .77]. The pitch shifting parameters for our manipulated stimuli assumed that the average female native Dutch speaker would have an F0 baseline around 210 Hz. This assumption was confirmed as participants’ mean F0 baseline was within 1 Hz of this value (i.e., 210.17 Hz), and baselines across both groups were normally distributed according to a Shapiro–Wilk test (*W* = 0.95, *p* = .16).

##### Addressing Possible Confounds

*Possible Lombard Speech in the Synchronous Speech Task*. Detriments to listening quality, such as the presence of another voice or background noise, can result in articulatory adjustments often referred to as “Lombard speech” (Garnier et al., 2010; Lombard, 1911). These adjustments often include an increase in intensity and F0, which encouraged us to confirm that such adjustments did not confound our critical F0 measure. The presence of a bidirectional convergence effect was the first indicator of a lack of Lombard Speech in the Synchronous Speech Task. That is, 11 out of 16 participants in the low F0 condition, as well as two participants in the high F0 condition who had an F0 even higher than the manipulated target, lowered their F0 toward the target despite the presence of another talker. Furthermore, we did not observe any reliable correlation between intensity and F0, *r*(62) = −.66, *p* = .51.

*An Effect of Synchronous Speech Performance*. Participants were explicitly instructed to converge to the model talker’s speech rate while our interest actually lied in F0. We conducted supplementary analyses to ensure that participants’ speech rate synchrony performance did not affect our critical measures. Crucially, one linear mixed effects model revealed a negligible effect (i.e., smaller than 1 Hz) of synchrony performance (as measured by trial-averaged temporal differences in word boundaries between the model talker and the participant) on raw F0, and another revealed no significant effect of temporal synchrony on the probability of convergence for a given trial. Details about these analyses are available on OSF (“analyses/sync_performance”).

### Discussion

Experiment 1 was primarily a stepping stone for the rest of the experiment series, and an adaptation of parts of the methodology of Bradshaw and McGettigan (2021) to Dutch. There were a number of assumptions inherent in the methodology of Experiment 1. Based on the secondary analyses discussed above, we have confirmed these assumptions with the current dataset. As predicted, the F0 of the model talker significantly predicted participants’ F0 in the synchronous speech task across both groups. The proportion of convergers in our sample (i.e., 75%) was noticeably higher than the proportion reported by Bradshaw and McGettigan (i.e., 60%), potentially due to the use of the pre-tested 210 Hz reference value for acoustic manipulations. Given that synchrony performance was not a significant factor in predicting participants’ convergence to F0, we attribute the reliable, bi-directional changes in participants’ mean F0 (i.e., per participant group) over the experiment to our acoustic manipulations and the synchronous speech paradigm. It is worth noting that the effect sizes in Figure 1B are conservative since they are based on analyses that are blind to the orientation of participants’ baselines and targets. For instance, there were participants who were assigned to the High F0 group but had an F0 even higher than that of the model talker. For these participants, convergence would mean going down in F0. Despite the presence of such participants in both groups, the between-group difference remained significant. While participants’ gradual exposure to the model talker over the course of the synchronous speech task would have resulted in the encoding of the model talker’s F0 (among other speech features) to some extent, Experiment 1’s design did not have a control condition that could measure possible effects of increasing familiarity with the talker. Thus, the next step was to provide reliable F0 information to participants prior to the synchronous speech task to observe its potential effects.

## Experiment 2 – Congruent Talker Information

Experiment 2 built upon the structure of Experiment 1 to implement an exposure phase between the reading and synchronous speech tasks, allowing for comparisons between baseline (reading) and synchronous speech F0s after providing participants with 20 min of controlled input at a pre-determined mean F0. Implementing talker familiarity into the design was crucial for our aim of investigating its possible impact on language production, and as a first step, we added an exposure task that provided congruent talker information to all participants across the two F0 groups. Once again, we expected the majority of the sample to display convergence to the model talker’s F0.

### Method

#### Participants

Participant selection in Experiment 2 was subject to the same criteria as Experiment 1. Another group (i.e., participants who had not participated in the previous experiment) of 32 native female Dutch speakers (age range: 18–33, *M*_age_ = 22.72, *SD*_age_ = 4.05) participated in Experiment 2, and they were randomly assigned to high and low F0 exposure groups.

#### Materials

The materials used in the reading and synchronous speech tasks of Experiment 2 were identical to Experiment 1. However, additional materials were used for the exposure task. These consisted of 10 2-min audio snippets about “everyday topics” such as news reports, animal fun facts, food recipes, etc. These snippets were recorded with the same model talker and manipulated to have the mean F0 of the high and low target values used in Experiment 1 (i.e., ±2 st from 210 Hz). Thus, a total of 20 manipulated sound files (see “stimuli” in OSF) were generated. In addition, one true-false question per text was generated to assess participants’ attention during the exposure task.

#### Procedure

Experiment 2 began with the same introductory procedure and reading task as Experiment 1. Following the reading task, participants performed the exposure task, in which they listened to the ten 2-min snippets of the model talker at a high or low F0 per their F0 group. After each snippet, participants were asked to respond to its true-false question. Although participants were not informed that their data would be excluded if they failed to respond to 80% of these questions correctly, the experiment instructions remarked that they were “expected to be able to answer at least 80%.” After listening to the snippets, participants moved onto the practice and synchronous speech tasks.

Participants heard the model talker at high or low F0 in the synchronous speech task per their F0 group. Thus, throughout the experiment, participants heard the model talker at only one mean F0 condition.

### Results

**Figure 2.**
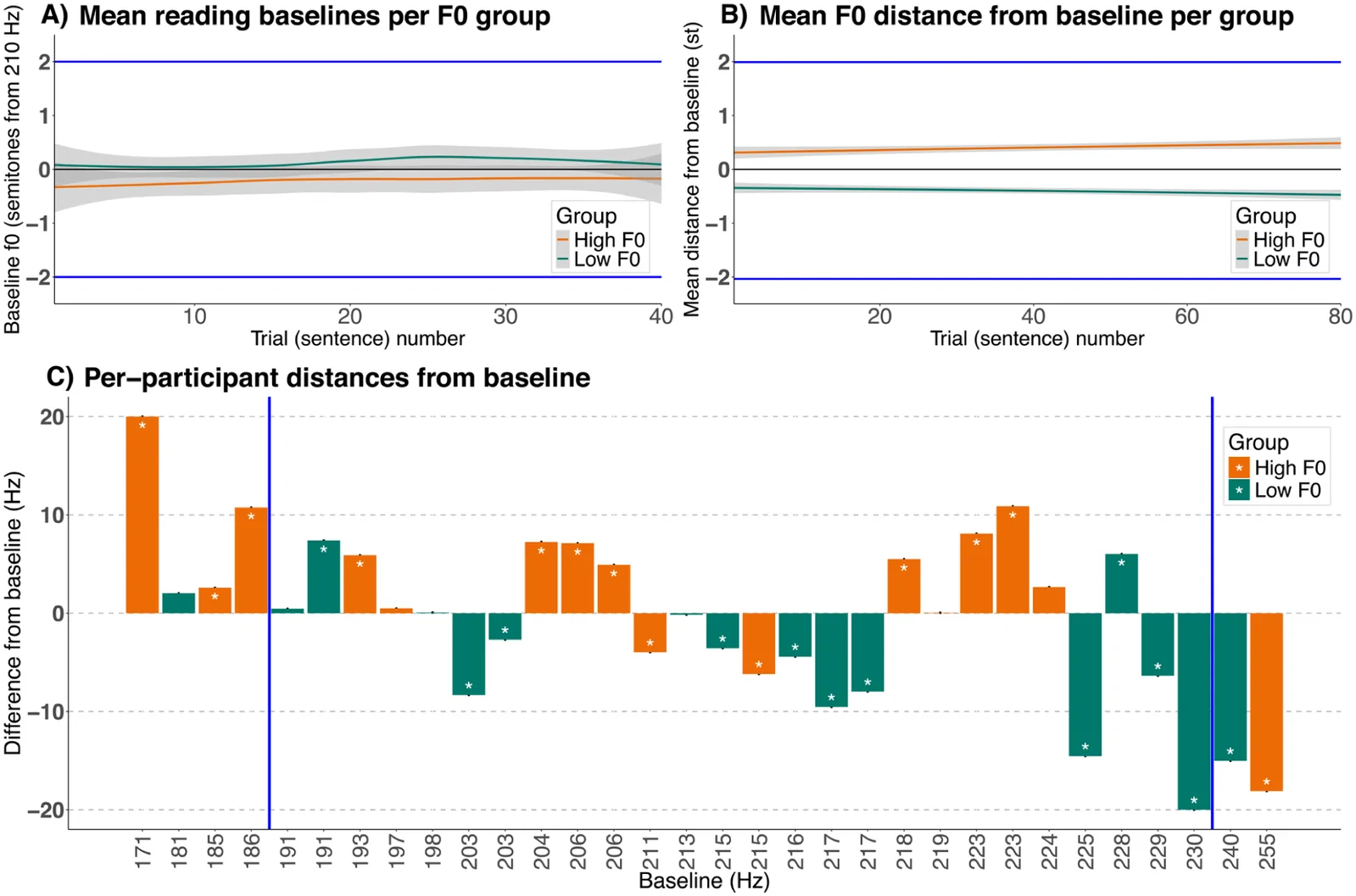
Reading F0, synchronous speech F0, and convergence metrics for Experiment 2. (A) Mean reading baselines per F0 group. (B) Mean F0 distance from baseline per group. (C) Per-participant distances from baseline. *Note.* Panel A demonstrates the comparable baselines across the two F0 groups in Experiment 2. On average, participants from both the high F0 and low F0 groups had baselines within a few Hz of the pre-tested mean of 210 Hz. Panel B demonstrates the changes in F0 in the synchronous speech task in Experiment 2. Here, the values are relative to each participant’s baseline, allowing for an overview of participant-normalized measures of changes in the direction of the model talker. Finally, Panel C displays convergence metrics on a by-participant basis. The *x* axis is ordered by participant baseline, but not scaled. Thus, each bar corresponds to a participant, with F0 group indicated by bar color (i.e., orange => high F0, green => low F0). The two vertical blue lines indicate the model talkers’ mean F0 values for the two F0 conditions (i.e., ~187 and ~236 Hz for the low and high F0 conditions, respectively). Bars with asterisks mark participants whose F0 changes from reading to synchronous speech were significant. In Experiment 2, based on the criteria described below, 21 out of the 32 participants converged.

All analyses, unless otherwise noted, were conducted using R (R Core Team, 2022). We used the same method of F0 extraction and analysis in Experiment 2 as in Experiment 1. A summary of the results is visualized in Figure 2. In Experiment 2, we aimed to gather data that could uncover whether the probability or magnitude of convergence would change (relative to Experiment 1) on account of the talker familiarity manipulation. While Experiment 2 is shaped by these cross-experimental expectations, comparisons between all three experiments are only reported after the results of Experiment 3.

#### Analyses of Convergence

##### Modeling Convergence in Raw F0 Across Tasks

We first modeled F0 across the two tasks using the same model and configuration as in Experiment 1. Within The Low Model, Task significantly predicted F0 (β = −4.94, *SE* = 0.38, *z* = −12.90, *p* < .001) whereas Condition did not (β = −3.79, *SE* = 5.02, *z* = −0.76, *p* = .46), meaning that participants’ F0s were comparable at reading when comparing the two groups, but significantly lower in the synchronous speech task (for the low F0 group). In addition, we observed a significant interaction between Task and Condition (β = 9.53, *SE* = 0.54, *z* = 17.60, *p* < .001). We re-leveled the dummy-coded model to have the high F0 synchronous speech condition on the intercept. Within this model (The High Model), Task significantly predicted higher F0 (β = 4.59, *SE* = 0.38, *z* = 11.99, *p* < .001) while the results for Condition and the interaction between Task and Condition were identical to those of The Low Model in all aspects except sign. This result indicated that participants in the high F0 group also converged to the model talker’s F0. Thus, like in the previous experiment, participants in Experiment 2 reliably converged to the model talker’s F0. Output tables for these models are available in Appendix B (Supplemental Material) and on OSF (under “analyses/exp_2”).

##### Within-Subject Analyses of Convergence

We used participant-by-participant ANOVAs to divide the sample into convergers and non-convergers. Based on the criteria described in Experiment 1, 21 participants converged, 4 diverged, and 7 displayed no significant differences in their F0 production across tasks.

#### Secondary Analyses

##### Baseline Homogeneity and Representativeness Across Groups

In addition to the results of The Low Model, a one-way ANOVA across all reading task trials revealed no significant differences in baseline F0 measures between the two F0 groups [*F*(1, 30) = 0.35, *p* = .56]. Participants’ mean F0 baseline was within 1 Hz of our pre-tested value of 210 Hz (i.e., 210.33 Hz), and baselines across both groups were normally distributed according to a Shapiro-Wilk test (*W* = 0.98, *p* = .94).

##### Possible Lombard Speech in the Synchronous Speech Task

In Experiment 2, like in Experiment 1, we found evidence for convergence in participants who were exposed to a low F0 talker and performed the synchronous speech task with a low F0 model talker. This already argues against the presence of confounds with Lombard effects in our data. In addition, we found no significant correlation between intensity and F0 across tasks, *r*(62) = .004, *p* = .98.

## Experiment 3 – Conflicting Talker Information

In Experiment 2, we successfully implemented an exposure phase into the methodology of Experiment 1, and observed that the majority of the sample (i.e., ~62%) still converged. This exposure-test paradigm only implemented congruent talker information. If a participant had heard the model talker at high F0 during exposure, they kept hearing her at the same average F0 in the synchronous speech task. This was less likely to lead to conflict in the perceptual stream compared to a scenario in which talker information conflicted with novel input. Thus, in line with our broader questions about the potential impact of talker representations on speech production and its implications regarding the use of shared representations, we ran a third experiment where participants received talker information during exposure, which was incongruent with the speech they encountered later in the synchronous speech task. In this configuration, conflict in the perceptual stream (due to expectancy violations about the talker’s F0) could manifest as a detriment to convergence.

### Method

#### Participants

Participant selection in Experiment 3 was subject to the same criteria as Experiment 1. A new set of 32 native female Dutch speakers (age range: 19–29, *M*_age_ = 23.25, *SD*_age_ = 2.60) participated in Experiment 3, and they were randomly assigned to high and low F0 exposure groups.

#### Materials

The materials used in Experiment 3 were identical to those used in Experiment 2.

#### Procedure

The experimental procedure of Experiment 3 was identical to that of Experiment 2 with one critical change: In Experiment 3, between the practice task and synchronous speech task, the mean F0 of the model talker was reversed. Thus, participants who were familiarized with the model talker at high F0 heard her at low F0 during the synchronous speech task, and vice versa. It is worth noting that the group labels for Experiment 3 reflect the F0 encountered in synchronous speech (i.e., a participant in the “high F0” group was exposed to low F0 and performed the synchronous speech task with high F0).

The practice task was identical to that of Experiment 1. Crucially, the mean F0 of the model talker was identical to the mean F0 each participant had been familiarized with in the exposure task. However, once participants moved onto the synchronous speech task, the F0 of the model talker was now reversed. That is, participants who were familiarized with the talker at high F0 now heard her at low F0, and vice versa. Besides this notable change, the synchronous speech task and the questionnaire/debriefing phases of Experiment 2 were identical to that of Experiment 1.

### Results

**Figure 3.**
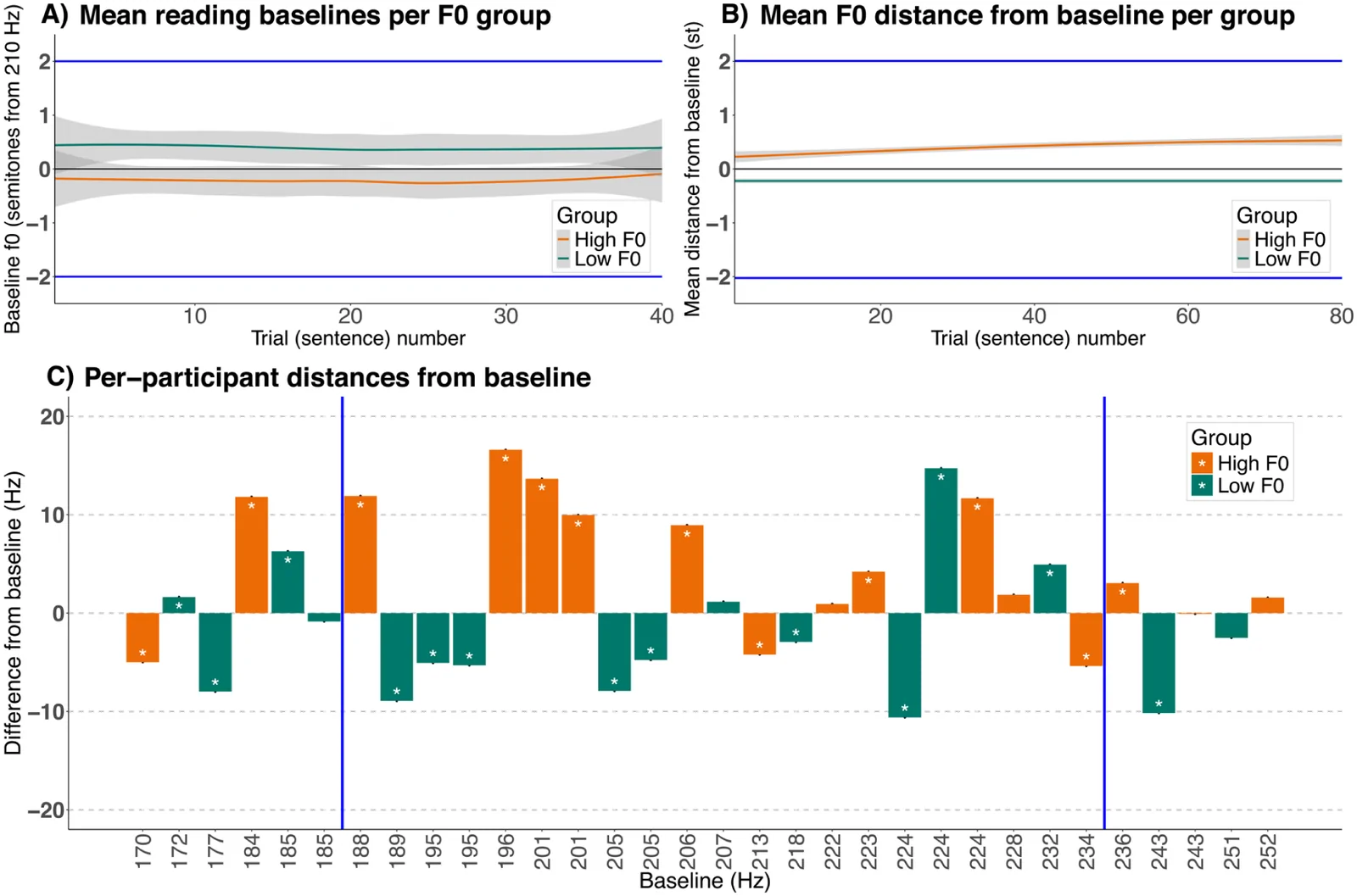
Reading F0, synchronous speech F0, and convergence metrics for Experiment 3. (A) Mean reading baselines per F0 group. (B) Mean F0 distance from baseline per group. (C) Per-participant distances from baseline. *Note*. Panel A demonstrates the comparable baselines across the two F0 groups in Experiment 3 (although the lines are farther apart than in previous experiments, the error ribbons are also larger, and the numerical difference is not statistically reliable). The mean F0 baseline across both groups was within a few Hz of the pre-tested mean of 210 Hz. Panel B demonstrates the changes in F0 in the synchronous speech task in Experiment 3. The values are relative to each participant’s baseline, allowing for an overview of participant-normalized measures of changes in the direction of the model talker. For this figure in particular, it is worth emphasizing that the black lines in Panels A and B represent different values. The black line in Panel A is the 210 Hz reference point, which was the basis for the acoustic manipulations. The black line in Panel B is normalized such that the colored lines indicate average distance from participants’ baselines. Panel C displays convergence metrics on a by-participant basis. The *x* axis is ordered by participant baseline, but not scaled. Thus, each bar corresponds to a participant, with F0 group indicated by bar color (i.e., orange => high F0, green => low F0; these labels refer to the F0 encountered in synchronous speech). The two vertical blue lines indicate the model talkers’ mean F0 values for the two F0 conditions (i.e., ~187 and ~236 Hz for the low and high F0 conditions, respectively). Bars with asterisks mark participants whose F0 changes from reading to synchronous speech were significant. In Experiment 3, based on the criteria described below in the main text, 18 out of the 32 participants converged.

All analyses, unless otherwise noted, were conducted using R (R Core Team, 2022). We used the same method of F0 extraction and analysis in Experiment 3 as in Experiment 1. In Experiment 3, out of 2,560 synchronous speech trials, only 8 were excluded from analyses due to response times shorter than 2,500 ms. A summary of results is visualized in Figure 3.

#### Analyses of Convergence

##### Modeling Convergence in Raw F0 Across Tasks

We first modeled F0 across the two tasks using the same model and configuration as in Experiments 1 and 2. The model was unchanged since the conditions in Experiment 3 were still made up of two perfectly anticorrelated components (i.e., participants with high talker F0 familiarization always heard low synchronous speech F0, and participants with low talker F0 familiarization always heard high synchronous speech F0).

Within The Low Model, Task significantly predicted F0 (β = −2.39, *SE* = 0.32, *z* = −7.55, *p* < .001) whereas Condition did not (β = 7.30, *SE* = 7.83, *z* = 0.93, *p* = .36). Thus, participants in the low F0 group significantly lowered their F0 in the synchronous speech task relative to the reading task, and the two F0 groups’ baselines were comparable in the reading task. In addition, we observed a significant interaction between Task and Condition (β = 7.49, *SE* = 0.45, *z* = 16.69, *p* < .001). Once again, we re-leveled the dummy-coded model to have the high F0 synchronous speech condition on the intercept. Within this model (The High Model), Task significantly predicted higher F0 (β = 5.09, *SE* = 0.32, *z* = 16.06, *p* < .001) while the results for Condition and the interaction between Task and Condition were identical to those of The Low Model in all aspects except sign. Thus, similar to Experiments 1 and 2, participants in both groups converged toward the target pitches in the synchronous speech tasks of their respective conditions in Experiment 3. Output tables for these models are available in Appendix C (Supplemental Material) and on OSF (under “analyses/exp_3”).

##### Within-Subject Analyses of Convergence

We used participant-by-participant ANOVAs to divide the sample into convergers and non-convergers. Convergence was assessed on the basis of the same three criteria as were used in Experiment 1. Based on these criteria, 18 participants converged, 7 diverged, and 7 displayed no significant differences in their F0 production across tasks.

#### Secondary Analyses

##### Baseline Homogeneity and Representativeness Across Groups

In addition to the results of The Low Model, a one-way ANOVA across all reading task trials revealed no significant differences in baseline F0 measures between the two F0 groups [*F*(1, 30) = 0.79, *p* = .38]. Participants’ mean F0 baseline was within 1 Hz of our pre-tested value of 210 Hz (i.e., 210.18 Hz), and baselines across both groups were normally distributed according to a Shapiro-Wilk test (*W* = 0.97, *p* = .48).

##### Possible Lombard Speech in the Synchronous Speech Task

In Experiment 3, we found no correlation between intensity and F0, *r*(62) = .04, *p* = .73. Additionally, we once again observed reliable convergence within the low F0 group.

## Cross-Experiment Analyses

We conducted a range of cross-experiment analyses to be able to measure the effects of talker familiarity (i.e., both congruent and conflicting talker information) on convergence. We measured the potential impact of congruent and conflicting talker information through comparisons of the convergence magnitude and the probability of convergence across all three experiments.

The dependent variables used in these cross-experiment analyses differed from those used in the within-experiment analyses. Notably, the relative/normalized variables used to measure convergence in the cross-experiment analyses took into account each participant’s baseline and the model talker’s target F0 for each trial to allow for cross-experimental comparisons.

### The Probability of Convergence

We first tested for the potential role of talker information on the probability of converging in a given trial. Here, we defined trials featuring convergence as those in which participants produced F0 values that fell between their baselines and the model talker’s target F0, with a 5% *leeway* to accommodate trials in which participants’ F0 values may have landed just beyond the target. It is important to remark that this percentage was calculated in semitone space as a proportion of the total distance between a participant’s baseline and their F0 target, and that additional analyses using 0% and 10% leeway led to convergent results, as reported in Supplemental Material (see “joint_analysis” on OSF).

**Figure 4.**
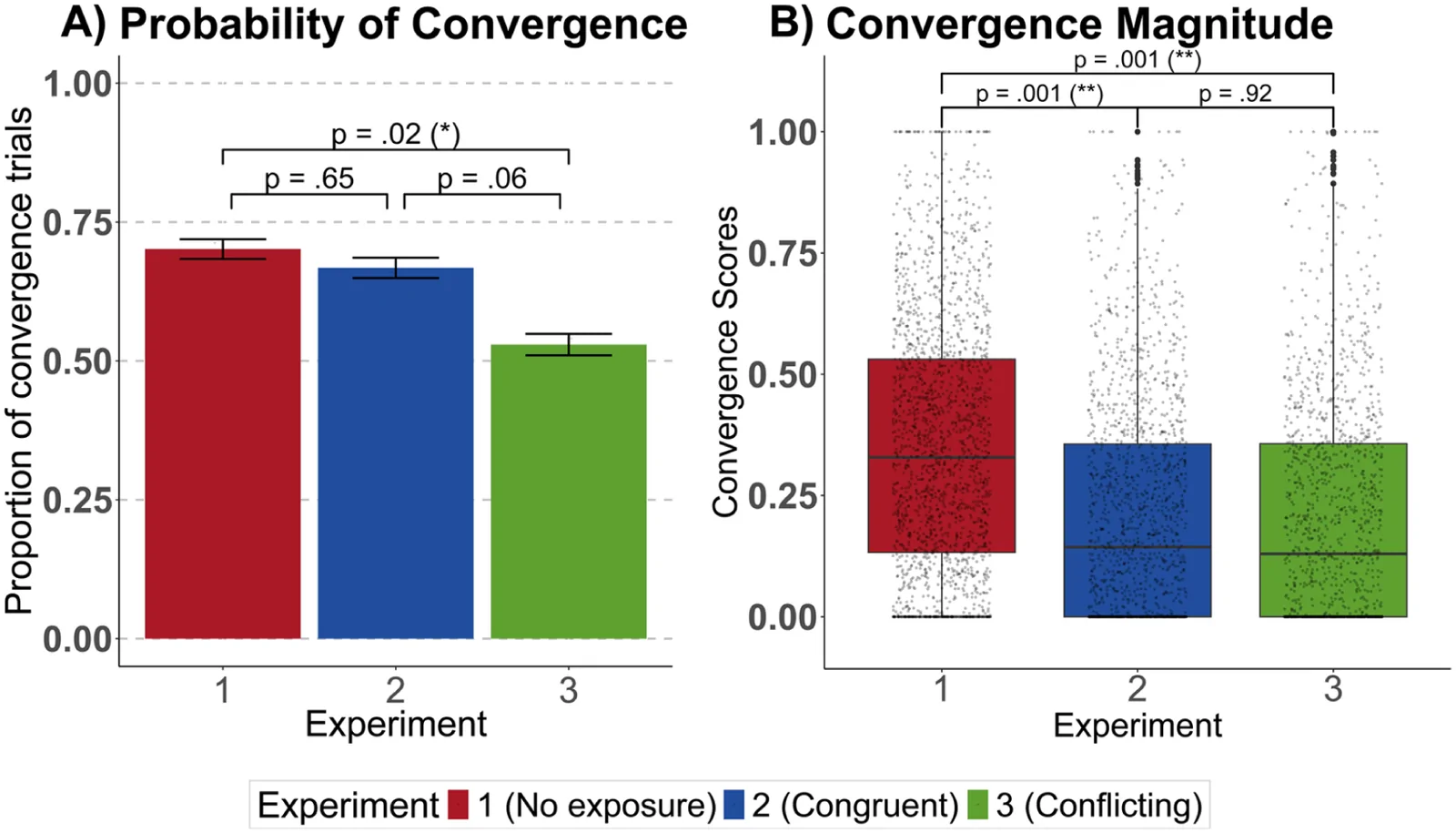
Probability of convergence and convergence scores across experiments. (A) Probability of convergence. (B) Convergence magnitude. *Note.* Figures display the results of cross-experiment comparisons. In both plots, data from Experiment 1 function as reference points. Panel A demonstrates the proportion of convergence trials within all trials across the three experiments on the *Y* axis and the experiment number on the *X* axis. With Experiment 1 as reference, while the congruent talker information in Experiment 2 does not lead to a significantly smaller probability of convergence, the conflicting talker information in Experiment 3 does. Panel B displays average convergence scores across the three experiments in combination with a scatter plot, which displays individual trial scores. The scores that could only have a value between 0 and 1 are on the *Y* axis, and the experiment number is on the *X* axis. For the purposes of visualization alone, non-convergence trials per the convergence score variable (i.e., trials with a score of 0) have been removed from the visualization (i.e., these trials were included in the analyses). Unlike the probability of convergence, the magnitude of convergence is significantly lowered by both congruent and conflicting talker information in Experiments 2 and 3, respectively.

We ran a generalized linear mixed effects model with Experiment as a fixed factor, with random intercepts for Participant ID and Item (i.e., sentence). The dependent variable was TrialConv (i.e., “trial convergence”; binary coded based on the criteria described above). Across 7,669 trials from 96 participants, the model intercept revealed that 66.8% of the trials in Experiment 1 featured convergence (β = .70, *SE* = 0.05, *z* = 13.58, *p* < .001; note that effect sizes do not directly reflect convergence trial proportions in logit space). Crucially, we found a significant reduction in the proportion of convergence trials in Experiment 3 which featured conflicting talker information (β = −.17, *SE* = 0.07, *z* = −2.34, *p* = .02), but no significant reduction in Experiment 2 which featured congruent talker information (β = −.03, *SE* = 0.07, *z* = −0.46, *p* = .65). A releveled model with Experiment 3 on the intercept revealed that the difference between Experiment 2 and Experiment 3 was also not significant (β = .14, *SE* = 0.07, *z* = 1.91, *p* = .06). Output tables for these two primary models are available in Appendix D (Supplemental Material) as well as on OSF (“analyses/joint_analyses”). A modified version of the initial model which also included Trial (i.e., Trial number; *z*-scored using the scale() function in R) as a fixed factor with an interaction term and a by-participant random slope did not reveal a trial effect or significant interactions. A final maximalist version of the model also included Encountered (i.e., a variable encoding whether a participant had encountered the sentence assigned to a trial in the reading task) as another fixed factor with an interaction term. This model did not include a random slope for Encountered due to convergence issues, but kept the by-participant random slopes for Trial. Once again, we only found a significant effect of Experiment 3 (i.e., conflicting talker information). These analyses are available on OSF (see “joint_analysis”), but are not reported here due to a lack of significant effects or interactions. Taken together, these results indicate that the conflicting talker information in Experiment 3 led to a significantly smaller proportion of convergence trials compared to the congruent talker information in Experiment 2 (see Figure 4).

### Convergence Magnitude

We conducted additional cross-experiment analyses that were restricted to the trials from participants that we had categorized as convergers within each experiment. This resulted in a subset of 4,951 trials from 62 participants (i.e., ~65% of all data). These participants were remarkably evenly distributed across the context F0 conditions with 2,471 high F0 context trials and 2,480 low F0 context trials (see Table 1). Given that trials from non-converging participants often did not feature any convergence at all, this subsetting of the data was carried out to obtain optimal estimates of convergence magnitudes across the three experiments.

**Table 1.** Number of Converging Participants and Convergence Trials Across Experiments and F0 Contexts.

Experiment	Experiment 1		Experiment 2 (Congruent)		Experiment 3 (Conflicting)	
F0 (Sync)	Low F0	High F0	Low F0	High F0	Low F0	High F0
Converged	12	12	10	11	10	8
Diverged	1	1	2	2	3	4
Stayed	3	3	4	3	3	4
% Convergence trials	69	71	64	69	49	56

The dependent variable for convergence magnitude was a single numeric value that constituted a participant’s *convergence score* on a given trial. To compute this score, we first calculated the distance between the participant’s baseline and the target F0 assigned to their participant group. This provided a numeric measure of the F0 *convergence space* that participant could potentially traverse. If a participant’s F0 in a given trial was between their baseline and the target, their convergence score was equal to the ratio of the F0 change from baseline to the baseline-target distance. For instance, a participant who went up by 0.6 st for a target 1.2 st above their baseline would have a convergence score of 0.5. If a participant’s F0 changed in the opposite direction, away from the target, then their convergence score would be 0 for that trial. This was on account of our theoretical assumption that participants were behaving qualitatively differently when converging as opposed to diverging or staying, and that a divergence measure (e.g., encoded as negative convergence) would therefore be incomparable to convergence scores. If a participant’s F0 changed in the direction of the target but overshot the target, their convergence score for that trial was inverse, with a cap at 200% of the baseline-target distance. Thus, a participant who went up by 1.2 st in the direction of a target that was 0.8 st higher than their baseline would have a convergence score of 0.5. Finally, we implemented a leeway similar to that used in the cross-experiment analyses for the probability of convergence. With a 5% leeway, trials with F0 values falling between 95% and 105% of the baseline-target distance were assigned a perfect convergence score of 1, and the inverse weighing of overshot trials began at 105% of the baseline-target distance. Participants whose target-to-baseline distance was within leeway bounds were assigned 0 convergence score on all trials since this would mean that their baselines are likely too close to the target for a reliable detection of convergence. We note that additional versions of these analyses with 0% and 10% leeway are also available in Supplemental Material (and can be customized further in R), with only numerical differences in outcomes.

We ran a linear mixed effects model on this dataset with Experiment as a fixed factor and with random intercepts for Participant ID. Random intercepts for Item (unique sentence IDs) were excluded due to convergence issues. The dependent variable was convergence score, described in the previous paragraph. Relative to Experiment 1 (β = .35, *SE* = 0.03, *z* = 12.56, *p* < .001), this model revealed a significant effect of both Experiment 2 (β = −.14, *SE* = 0.03, *z* = −3.35, *p* = .001) and Experiment 3 (β = −.14, *SE* = 0.04, *z* = −3.36, *p* = .001), indicating that both conflicting and congruent talker information led to a decline in the effect size of convergence score. Expanding the model to include Trial (trial number; *z*-scored) and encountered (i.e., binary variable encoding whether the sentence had been encountered at the reading task) did not yield additional significant effects, and the effects of Experiments 2 and 3 persisted (see OSF for these analyses).

There was empirical precedent for also considering the potential role of the convergence target’s distance from a participant’s baseline (e.g., Babel, 2012; Priva & Sanker, 2019). It is unreasonable to expect interlocutors to change their F0 too much if the target is too far, and it might be challenging for statistical models to detect convergence when the target is too close one’s baseline F0. For instance, Bradshaw and McGettigan (2021), whose results we replicated in Experiment 1, had to include an “extra-low F0” condition in their synchronous speech tasks after observing that their low F0 manipulated stimuli were still too high in relation to where the sample’s mean F0 baselines stood. Interestingly, they later found that the extra-low F0 condition led to a smaller convergence magnitude than the low F0 condition, presumably due to the increased distance from the typical participant’s F0 baseline. In line with this evidence, we expanded our model to include Baseline Distance with an interaction term, which encoded the (non-signed) semitone distance of the observed F0 of each trial relative to that participant’s F0 baseline. The model revealed no significant effect of Baseline Distance (β = −.03, *SE* = 0.02, *z* = −1.47, *p* = .14), and no significant interactions with Experiment 2 (β = .03, *SE* = 0.03, *z* = 1.01, *p* = .31) or Experiment 3 (β = −.02, *SE* = 0.03, *z* = 0.90, *p* = .37) while the effects of both experiments persisted. Thus, the results of the cross-experimental comparisons were not influenced by the distance between participants’ baselines and their F0 on a given trial.

Overall, these analyses suggested that the magnitude of participants’ convergence was similarly affected by the talker F0 information introduced in both Experiment 2 and Experiment 3. However, the probability of convergence was significantly lower in Experiment 3 (i.e., with conflicting talker information) but not in Experiment 2 (i.e., with congruent talker information).

## General Discussion

The series of experiments reported in this manuscript sought to investigate the potential role of talker representations in language production as evidenced by their impact on convergence to a model talker’s F0. In Experiment 1, participants in two F0 groups (i.e., high and low) performed a (baseline) reading task, followed by a synchronous speech task. Participants converged to the manipulated F0 of the model talker in both groups. Thus, Experiment 1 constituted a successful conceptual replication of Bradshaw and McGettigan (2021) in Dutch. It is worth noting that the proportion of convergers in our sample was larger in our study. We attribute these increases to tighter experimental control due to laboratory (as opposed to online) testing and to stimulus optimization (i.e., the reference mean F0 value of 210 Hz, and the 2 st F0 manipulation size) based on pre-testing. Overall, the first experiment established the validity of the synchronous speech paradigm for the purposes of the present study and laid the groundwork for the two following experiments.

In Experiment 2, we familiarized two groups of participants with either a high or a low F0 variant of the same model talker (i.e., using identical recordings that only differed in F0 manipulations). This familiarization involved a 20-min passive listening task between the baseline reading task and the synchronous speech task. Thus, participants only heard the model talker at one mean F0 level, and only heard her after providing us with their F0 baselines. Once again, we found evidence that a majority of the sample across both F0 groups reliably converged toward the model talker’s F0 in the synchronous speech task. Experiment 2’s results also provided the first point of comparison between Experiment 1’s *reference* results. Overall, we observed convergence in the majority of the sample across both talker F0 groups, and we detected a smaller magnitude of convergence compared to Experiment 1 (further discussion of cross-experiment differences can be found later in this manuscript following the results of Experiment 3). However, any answer to the larger question of whether talker representations are shared across perception and production would be incomplete without another scenario involving conflicting talker information.

To that end, in Experiment 3, we implemented the same methodology as Experiment 2 with one crucial change: In Experiment 3, we flipped the F0 condition between the exposure task and the synchronous speech task. Critically, in the latter task, the F0 of the sentences was *flipped* such that participants who were familiarized with the model talker at a low F0 would now hear her at a high F0, and vice versa. Thus, Experiment 3 allowed for direct comparisons between the effects of conflicting and congruent talker information with Experiment 1 as reference. We found evidence for convergence in both F0 groups. Crucially, in line with our hypothesis that talker representations are shared across perception and production, we had anticipated that this F0 flip and the resulting expectancy violations might lead to conflict in the perceptual stream, which in turn might manifest in production as hindered convergence. By both of our cross-experiment analysis metrics, convergence was hindered. However, the precise mechanism behind this hindrance cannot be specified given the limitations of our design: it may be that the observed hindrance of convergence was due to the conflicting F0 information in exposure versus synchronous speech, or perhaps due to increased input variability. Thus, we emphasize that our interpretation of the effects as arising from a “conflict in the perceptual stream” is speculative. Experiment 3 resulted in a significantly smaller probability of convergence across all trials compared to Experiment 1 while Experiment 2’s impact on the probability of convergence (i.e., the effect size of the model estimate) was not significant. In comparison to Experiment 1, the negative impact of conflicting talker information on convergence in both metrics indicates that at least some resources that encode talker information are shared across perception and production.

Overall, the implementation of the synchronous speech paradigm was successful in reliably observing convergence to the model talker’s speech. As explicitly confirmed by the post-experiment questionnaires (see OSF for output), out of 96 participants across the three experiments, none suspected that our interest might be in F0 rather than in speech rate or the ability to synchronize to the model talker’s word onsets or offsets. In all three experiments, more than half of the sample converged, and these convergers were evenly distributed across F0 groups. As evident in the significant differences between the group means across reading baselines and synchronous speech F0s in all three experiments, the general tendency among participants was to move in the direction that corresponds to their (synchronous speech) F0 group. For instance, participants in the high F0 groups, despite the presence of participants whose baselines were above the high F0 target, moved up from their baselines (at the group level) across all three experiments. Thus, our results support the assertion that convergence is a generally automatic process in terms of its implicit nature. The majority of participants in all three experiments converged to F0 without explicitly being instructed to do so. However, the lack of a significant difference between convergence magnitudes across Experiments 2 and 3, and the absence of a ceiling effect challenge accounts of fully automatic, *resource-free* convergence, and tighter integration between perception and production (e.g., Pickering & Garrod, 2013).

Our cross-experiment analyses revealed that the probability of convergence was significantly reduced in the presence of conflicting talker information (Experiment 3), but not in the presence on congruent talker information (Experiment 2). However, convergence magnitude, measured within the convergers across all three experiments, was comparable across Experiments 2 and 3 as both congruent and conflicting talker information led to a smaller convergence magnitude compared to Experiment 1. Given the well-established facilitatory role of talker familiarity in speech perception, we consider it unlikely that this may be a manifestation of increased perceptual conflict affecting speech production. Instead, we speculate that the reduction in convergence magnitude in Experiment 2 (relative to Experiment 1) is likely due to differences in the model talker’s speech across the exposure and synchronous speech materials. While we strictly controlled the model talker’s F0 across these sets of stimuli, other acoustic (as well as lexical, syntactic, and semantic) features of her speech were untouched in order to preserve the perceived naturalness of her voice. Consequently, these features of her speech could be considered *unfamiliar* for all participants, who would be exposed to the same differences between the exposure and synchronous speech tasks in Experiments 2 and 3. For instance, acoustic analyses of the model talker’s speech across the familiarization materials and the sentences used in the reading and synchronous speech tasks have revealed that she had a significantly higher speech rate (i.e., words per minute) in the reading/sync speech sentences (see OSF; “analyses/speech_rate”). This, in turn, may be responsible for the apparent hindrance of convergence even in the presence of F0-congruent talker information, reducing the magnitude of convergence across multiple acoustic variables.

Our results speak primarily to the nature of the phonetic level of convergence and to an ongoing debate regarding representational parity (see Gambi & Pickering, 2017 for a discussion of existing evidence) between perception and production. Both strands of our cross-experiment analyses revealed that talker representations, built up over 20 min of passive listening, are able to influence production. Furthermore, the effects we report likely reflect more than increased processing costs in the perceptual stream since our analyses were based on acoustic analyses rather than measures of processing time/cost (e.g., reaction times). In addition, by using acoustic measures only (i.e., without perceptual measures), we have ensured that what we measure and report pertains to modifications to the speech signal itself (i.e., as opposed to its perception by another group of raters). While frameworks which assume a tight link between perception and production (McQueen & Meyer, 2019; Pickering & Garrod, 2004) might therefore be sufficient to explain our pattern of results, it is also worth emphasizing that despite our tight control of the speech acoustics of all stimuli and the laboratory recording conditions, we cannot categorically rule out sociolinguistic influences on our data. For instance, participants may have made various assumptions about the model talker’s personality, physical features, sociability, etc., and these may be factors underlying part of the unsurprising individual variation we observed across all three experiments. Nevertheless, we re-emphasize that the F0 changes crucial to our study are parsimoniously explainable through accounts relying on priming, irrespective of any potential social motivation for participants’ convergence.

It is also worth noting how the methodological configuration of our experiments, our method of instruction, and the nature of the synchronous speech task may have affected our results. All three experiments included explicit instructions to converge to the temporal characteristics of the model talker’s speech, in order to conceal the aim of the experiments and our interest in the critical acoustic variable F0. One might speculate that this explicit instruction to convergence to temporal features may have affected convergence to other speech features as well, potentially including F0. However, as further supplementary within-experiment analyses show (see OSF under “analyses/sync_performance”), synchronous speech performance (as measured by average difference scores based on the model talker’s word boundaries) had no effect on the probability of trial-by-trial convergence, and only had a negligible impact on raw F0 measures. Though more traditional forward modeling-influenced accounts of convergence (e.g., Pickering & Garrod, 2004) would predict facilitation within (and potentially between) different levels, more recent evidence highlights that it is common for interlocutors to selectively converge both within and across levels (e.g., convergence to speech rate may not predict convergence to other suprasegmental features of speech; Ostrand & Chodroff, 2021).

It is important to recognize that many previous experiments which used convergence paradigms (e.g., Nielsen, 2011, 2014; Pardo et al., 2012, 2013, 2018; Schertz, 2025) tested the effect of talker information to some extent, as they often relied on repeated exposure to a talker’s speech either in a dedicated exposure phase or on a trial-by-trial basis. In doing so, they also tapped into the mechanisms that govern the interaction between production and perception, which we assume to be similar or identical to the mechanisms our participants relied on. A particular strength of our synchronous speech exposure-test methodology was the unification of multiple design choices that can be found across the literature. We had tight experimental control over speech acoustics across exposure and test in all three experiments. The three-second countdown in synchronous speech, combined with the long sentences we chose as stimuli, made it very challenging for participants to read and plan an F0 contour for the entire sentence. The instruction to converge temporally encouraged participants to continuously attend to the model talker’s speech as they believed their task was to synchronize to speech rate. Finally, the lack of explicit instruction to converge to F0 (or to “imitate speech” in general) increased the probability that our data resulted from automatic mechanisms, as hypothesized. Thus, while we do not claim that our methodology taps into different mechanisms than those observed in previous studies, we assert that it is more effective at catching the perception-production link in action.

One might conceptualize talker representations as part of the phonetic or phonological levels (i.e., containing talker-specific phonetic or phonological representations) in models of alignment (e.g., Pickering & Garrod, 2004) or as a set of algorithms and normalization processes that interact only with the phonetic or phonological levels and are selectively used depending on the talker. In either case, our analyses suggest that talker representations are shared between perception and production, functioning as the first filters of incoming speech (e.g., affecting how acoustic input is mapped onto phonemes by modifying weights in a speech recognition model) and the last filters of speech output (e.g., by modifying how the selected phoneme is mapped onto articulatory systems).

There are also limitations of our design worth discussing. For instance, our exposure phases in Experiments 2 and 3 were limited to 20 min of passive listening. This was primarily a methodological feasibility decision to allow Experiments 2 and 3 to fit within single hour-long experiment sessions. It is plausible that longer exposure durations might lead to a larger impact on production, although there is evidence that talker familiarity benefits for voice detection are observable with as little as 10 min of exposure, and that intelligibility benefits continue to build up until at least an hour (Holmes et al., 2021). Future research may, in particular, wish to incorporate multiple exposure phases in a multi-session design to provide a more ecologically valid view of familiarity over longer periods of time. Another limitation of the present study was a broader conceptual constraint that was necessitated by the talker information manipulation. Namely, we could only familiarize one participant with one mean F0 condition, with no potential *defamiliarization* method available. This resulted in between-experiment and between-participant analyses that featured extensive individual variation, though this limitation was in part addressed by appropriate random effect structures in our analyses. Finally, it is worth noting that our use of a single talker with two manipulated versions instead of multiple talkers was a deliberate design choice that aimed to maximize experimental control over stimulus acoustics. In addition, the identical non-F0 features of the model talker’s speech across the two F0 talker groups in Experiments 2 and 3 ensured that we could attribute any significant changes in convergence to effect of our F0 manipulation. An alternative to the impossible task of familiarizing a listener with more than one (opposite) within-talker acoustic manipulation condition could involve incorporating multiple talkers into a design in which participants are familiarized with different talkers to different extents. Such a design could shed light on the talker-specificity of the effects that we report here, and tease apart any global adjustments that may have affected all incoming speech in our design.

## Conclusion

Overall, our results indicate that, when convergence occurs, it leads to similar changes in one’s speech in terms of acoustics in the target speech feature irrespective of the congruence of talker information. Crucially, our findings suggest that the probability of convergence is negatively impacted only by the presence of conflicting talker information, when compared to a baseline case without familiarization. These results, in turn, provide evidence for representational parity between perception and production. That is, they show, as shown more indirectly before in exposure-test imitation or acoustic familiarity paradigms (M. Kim et al., 2011; Nielsen, 2011; Pardo et al., 2017; Pinget, 2022), that talker representations formed exclusively through perception can significantly affect one’s production. In addition, our results go beyond these prior studies by indicating not only convergence to a specific talker but also that convergence is modulated by the variability in that talker’s F0.

## Supplemental Material

sj-docx-1-qjp-10.1177_17470218261442873 – Supplemental material for The Role of Talker Information in Phonetic Convergence Supplemental material, sj-docx-1-qjp-10.1177_17470218261442873 for The Role of Talker Information in Phonetic Convergence by Orhun Uluşahin, Elhaam Parveen Hasan, Hans Rutger Bosker, Antje S. Meyer and James M. McQueen in Quarterly Journal of Experimental Psychology

## References

[bibr1-17470218261442873] Aubanel V. Nguyen N. (2020). Speaking to a common tune: Between-speaker convergence in voice fundamental frequency in a joint speech production task. PLoS ONE, 15(5), e0232209. 10.1371/journal.pone.0232209 PMC7197779 32365075

[bibr2-17470218261442873] Babel M. (2012). Evidence for phonetic and social selectivity in spontaneous phonetic imitation. Journal of Phonetics, 40(1), 177–189. 10.1016/j.wocn.2011.09.001

[bibr3-17470218261442873] Babel M. Bulatov D. (2012). The role of fundamental frequency in phonetic accommodation. Language and Speech, 55(2), 231–248. 10.1177/0023830911417695 22783633

[bibr4-17470218261442873] Bell L. Gustafson J. Heldner M. (2003). Prosodic adaptation in human-computer interaction. In Proceedings of ICPHS (Vol. 3, pp. 833-836). Citeseer.

[bibr5-17470218261442873] Boersma P. Weenink D. (2022). Praat: Doing phonetics by computer (Version 6.2.04) [Computer software]. http://www.praat.org/

[bibr6-17470218261442873] Bradshaw A. R. McGettigan C. (2021). Convergence in voice fundamental frequency during synchronous speech. PLOS ONE, 16(10), e0258747. 10.1371/journal.pone.0258747 PMC8530294 34673811

[bibr7-17470218261442873] Bradshaw A. R. Wheeler E. D. McGettigan C. Lametti D. R. (2025). Sensorimotor learning during synchronous speech is modulated by the acoustics of the other voice. Psychonomic Bulletin & Review, 32(1), 306–316. 10.3758/s13423-024-02536-x PMC11836077 38955989

[bibr8-17470218261442873] Branigan H. P. Pickering M. J. Cleland A. A. (2000). Syntactic co-ordination in dialogue. Cognition, 75(2), B13–B25. 10.1016/S0010-0277(99)00081-5 10771277

[bibr9-17470218261442873] Branigan H. P. Pickering M. J. McLean J. F. Cleland A. A. (2007). Syntactic alignment and participant role in dialogue. Cognition, 104(2), 163–197. 10.1016/j.cognition.2006.05.006 16876778

[bibr10-17470218261442873] Branigan H. P. Pickering M. J. Pearson J. McLean J. F. Brown A. (2011). The role of beliefs in lexical alignment: Evidence from dialogs with humans and computers. Cognition, 121(1), 41–57. 10.1016/j.cognition.2011.05.011 21723549

[bibr11-17470218261442873] Brennan S. E. Clark H. H. (1996). Conceptual pacts and lexical choice in conversation. Journal of Experimental Psychology: Learning, Memory, and Cognition, 22(6), 1482–1493. 10.1037/0278-7393.22.6.1482 8921603

[bibr12-17470218261442873] Cai Z. G. Sun Z. Zhao N. (2021). Interlocutor modelling in lexical alignment: The role of linguistic competence. Journal of Memory and Language, 121, 104278. 10.1016/j.jml.2021.104278

[bibr13-17470218261442873] Casasanto L. S. Jasmin K. Casasanto D. (2010). Virtually accommodating: Speech rate accommodation to a virtual interlocutor. In Ohlsson S. Catrambone R. (Eds.), Proceedings of the annual meeting of the cognitive science society (Vol. 32, pp. 127–132). Cognitive Science Society. https://escholarship.org/uc/item/3vg3g1ds

[bibr14-17470218261442873] Gambi C. Pickering M. J. (2017). Models linking production and comprehension. In Fernández E. M. Cairns H. S. (Eds.), The handbook of psycholinguistics (1st ed., pp. 157–181). Wiley. 10.1002/9781118829516.ch7

[bibr15-17470218261442873] Garnier M. Henrich N. Dubois D. (2010). Influence of sound immersion and communicative interaction on the lombard effect. Journal of Speech, Language, and Hearing Research, 53(3), 588–608. 10.1044/1092-4388(2009/08-0138) 20008681

[bibr16-17470218261442873] Garrod S. Anderson A. (1987). Saying what you mean in dialogue: A study in conceptual and semantic co-ordination. Cognition, 27(2), 181–218. 10.1016/0010-0277(87)90018-7 3691025

[bibr17-17470218261442873] Holmes E. Domingo Y. Johnsrude I. S. (2018). Familiar voices are more intelligible, even if they are not recognized as familiar. Psychological Science, 29(10), 1575–1583. 10.1177/0956797618779083 30096018

[bibr18-17470218261442873] Holmes E. To G. Johnsrude I. S. (2021). How long does it take for a voice to become familiar? Speech intelligibility and voice recognition are differentially sensitive to voice training. Psychological Science, 32(6), 903–915. 10.1177/0956797621991137 PMC13020942 33979256

[bibr19-17470218261442873] Kim C. S. Chamorro G. (2021). Nativeness, social distance and structural convergence in dialogue. Language, Cognition and Neuroscience, 36(8), 984–1000. 10.1080/23273798.2021.1916544

[bibr20-17470218261442873] Kim M. Horton W. S. Bradlow A. R. (2011). Phonetic convergence in spontaneous conversations as a function of interlocutor language distance. Laboratory Phonology, 2(1), 125–156. 10.1515/labphon.2011.004 PMC3638967 23637712

[bibr21-17470218261442873] Kuznetsova A. Brockhoff P. B. Christensen R. H. B. (2017). LmerTest Package: Tests in linear mixed effects models. Journal of Statistical Software, 82, 1–26. 10.18637/jss.v082.i13

[bibr22-17470218261442873] Levelt W. J. M. (1999). Models of word production. Trends in Cognitive Sciences, 3(6), 223–232. 10.1016/S1364-6613(99)01319-4 10354575

[bibr23-17470218261442873] Levelt W. J. M. Roelofs A. Meyer A. S. (1999). A theory of lexical access in speech production. Behavioral and Brain Sciences, 22(1), 1–38. 10.1017/S0140525X99001776 11301520

[bibr24-17470218261442873] Lombard E. (1911). Le signe de televation de la voix. [The sign of the rise of the voice] Annu. Maladies Oreille Larynx Nez Pharynx, 27, 101–119.

[bibr25-17470218261442873] McClelland J. L. Elman J. L. (1986). The TRACE model of speech perception. Cognitive Psychology, 18(1), 1–86. 10.1016/0010-0285(86)90015-0 3753912

[bibr26-17470218261442873] McQueen J. M. Meyer A. S. (2019). Key issues and future directions: Toward a comprehensive cognitive architecture for language use. In Hagoort P. (Ed.), Human language (pp. 85–96). The MIT Press. 10.7551/mitpress/10841.003.0009

[bibr27-17470218261442873] Nielsen K. (2011). Specificity and abstractness of VOT imitation. Journal of Phonetics, 39(2), 132–142. 10.1016/j.wocn.2010.12.007

[bibr28-17470218261442873] Nielsen K. (2014). Phonetic imitation by young children and its developmental changes. Journal of Speech, Language, and Hearing Research, 57(6), 2065–2075. 10.1044/2014_JSLHR-S-13-0093 25076096

[bibr29-17470218261442873] Norris D. McQueen J. M. (2008). Shortlist B: A Bayesian model of continuous speech recognition. Psychological Review, 115(2), 357–395. 10.1037/0033-295X.115.2.357 18426294

[bibr30-17470218261442873] Nygaard L. C. Pisoni D. B. (1998). Talker-specific learning in speech perception. Perception & Psychophysics, 60(3), 355–376. 10.3758/BF03206860 9599989

[bibr31-17470218261442873] Nygaard L. C. Sommers M. S. Pisoni D. B. (1994). Speech perception as a Talker-Contingent Process. Psychological Science, 5(1), 42–46. 10.1111/j.1467-9280.1994.tb00612.x PMC3081685 21526138

[bibr32-17470218261442873] Ostrand R. Chodroff E. (2021). It’s alignment all the way down, but not all the way up: Speakers align on some features but not others within a dialogue. Journal of Phonetics, 88, 101074. 10.1016/j.wocn.2021.101074 PMC8345023 34366499

[bibr33-17470218261442873] Pardo J. S. Gibbons R. Suppes A. Krauss R. M. (2012). Phonetic convergence in college roommates. Journal of Phonetics, 40(1), 190–197. 10.1016/j.wocn.2011.10.001

[bibr34-17470218261442873] Pardo J. S. Jordan K. Mallari R. Scanlon C. Lewandowski E. (2013). Phonetic convergence in shadowed speech: The relation between acoustic and perceptual measures. Journal of Memory and Language, 69(3), 183–195. 10.1016/j.jml.2013.06.002

[bibr35-17470218261442873] Pardo J. S. Urmanche A. Wilman S. Wiener J. (2017). Phonetic convergence across multiple measures and model talkers. Attention, Perception, & Psychophysics, 79(2), 637–659. 10.3758/s13414-016-1226-0 27826946

[bibr36-17470218261442873] Pardo J. S. Urmanche A. Wilman S. Wiener J. Mason N. Francis K. Ward M. (2018). A comparison of phonetic convergence in conversational interaction and speech shadowing. Journal of Phonetics, 69, 1–11. 10.1016/j.wocn.2018.04.001

[bibr37-17470218261442873] Pickering M. J. Garrod S. (2004). Toward a mechanistic psychology of dialogue. Behavioral and Brain Sciences, 27(2), 169–190. 10.1017/S0140525X04000056 15595235

[bibr38-17470218261442873] Pickering M. J. Garrod S. (2013). An integrated theory of language production and comprehension. Behavioral and Brain Sciences, 36(4), 329–347. 10.1017/S0140525X12001495 23789620

[bibr39-17470218261442873] Pinget A.-F. (2022). Individual differences in phonetic imitation and their role in sound change. Phonetica, 79(5), 425–457. 10.1515/phon-2022-2026 36395076

[bibr40-17470218261442873] Priva U. C. Sanker C. (2019). Limitations of difference-in-difference for measuring convergence. Laboratory Phonology, 10(1), Article 1. 10.5334/labphon.200

[bibr41-17470218261442873] R Core Team. (2022). R: A language and environment for statistical computing. R Foundation for Statistical Computing. https://www.R-project.org/

[bibr42-17470218261442873] Rahimi Z. Kumar A. Litman D. Paletz S. Yu M. (2017). Entrainment in multi-party spoken dialogues at multiple linguistic levels. Interspeech, 2017, 1696–1700. 10.21437/Interspeech.2017-1568

[bibr43-17470218261442873] Reinisch E. (2016). Speaker-specific processing and local context information: The case of speaking rate. Applied Psycholinguistics, 37(6), 1397–1415. 10.1017/S0142716415000612

[bibr44-17470218261442873] Schertz J. (2025). Individual uniformity in phonetic imitation: Assessing the stability of individual variability across features and tasks. Journal of Phonetics, 108, 101376. 10.1016/j.wocn.2024.101376

[bibr45-17470218261442873] Schultz B. G. O’brien I. Phillips N. McFarland D. H. Titone D. Palmer C. (2016). Speech rates converge in scripted turn-taking conversations. Applied Psycholinguistics, 37(5), 1201–1220. 10.1017/S0142716415000545

[bibr46-17470218261442873] Schweitzer A. Lewandowski N. (2014). Social factors in convergence of F1 and F2 in spontaneous speech. In Fuchs S. Mücke D. Lancia L. Hermes A. Grice M. (Eds.), Proceedings of the 10th international seminar on speech production, ISSP 2014 (pp. 391–394). https://www.scopus.com/inward/record.uri?eid=2-s2.0-85039164612&partnerID=40&md5=f2bfd3147d28baabd4e03c1b7891ecea

[bibr47-17470218261442873] Trainin N. Shetreet E. (2024). “Wait, how did you call this?”: Speaker-specific word choices are stored and generalized. Journal of Experimental Psychology: Learning, Memory, and Cognition, 51(2), 320–335. 10.1037/xlm0001348 38900548

[bibr48-17470218261442873] Zhang X. Holt L. L. (2018). Simultaneous tracking of coevolving distributional regularities in speech. Journal of Experimental Psychology: Human Perception and Performance, 44(11), 1760–1779. 10.1037/xhp0000569 PMC6205888 30272462

