# Supplementary material for: The Role of Talker Information in Phonetic Convergence
Source: Q J Exp Psychol (Hove). 2026 Apr 3;79(10):2740–57. doi: 10.1177/17470218261442873 (PMC13601685; doi:10.1177/17470218261442873)
Supplement: Supplementary file 1 [file sj-docx-1-qjp-10.1177_17470218261442873.docx]

Supplementary Material for:

**The Role of Talker Information in Phonetic Convergence**

Orhun Uluşahin^1,2^, Elhaam Parveen Hasan^3^, Hans Rutger Bosker^4,1^, Antje S. Meyer^1,4^ and James M. McQueen^4,1^

^1^ Max Planck Institute for Psycholinguistics, Nijmegen, NL

^2^ International Max Planck Research School for Language Sciences, Nijmegen, NL

^3^ Faculty of Science, Radboud University, Nijmegen, NL

^4^ Donders Centre for Cognition, Radboud University, Nijmegen, NL

**Author Note**

Orhun Uluşahin <https://orcid.org/0009-0006-3703-0272>

Elhaam Parveen Hasan

Hans Rutger Bosker <https://orcid.org/0000-0002-2628-7738>

Antje S. Meyer <https://orcid.org/0000-0002-7735-9025>

James M. McQueen <https://orcid.org/0000-0003-3734-6286>

Correspondence concerning this article should be addressed to Orhun Uluşahin, Donders Centre for Cognition, Thomas Van Aquinostraat 4, 6525GD Nijmegen, The Netherlands. Email: [orhun.ulusahin@donders.ru.nl](mailto:orhun.ulusahin@donders.ru.nl)

**Appendix A**

**Model Outputs from Experiment 1**

**“Low Model”**

**“High Model”**

**Appendix B**

**Model Outputs from Experiment 2**

**“Low Model”**

**“High Model”**

**Appendix C**

**Model Outputs from Experiment 3**

**“Low Model”**

**“High Model”**

**Appendix D**

**Model Outputs of Cross-Experiment Analyses**

**Probability of Convergence**

**Magnitude of Convergence**
